# Proteomic Differences between Male and Female Anterior Cruciate Ligament and Patellar Tendon

**DOI:** 10.1371/journal.pone.0096526

**Published:** 2014-05-12

**Authors:** Dianne Little, J. Will Thompson, Laura G. Dubois, David S. Ruch, M. Arthur Moseley, Farshid Guilak

**Affiliations:** 1 Department of Orthopaedic Surgery, Duke University Medical Center, Durham, North Carolina, United States of America; 2 Proteomics Core Facility, Institute for Genome Science & Policy, Duke University Medical Center, Durham, North Carolina, United States of America; 3 Department of Biomedical Engineering, Duke University Medical Center, Durham, North Carolina, United States of America; University of Rochester, United States of America

## Abstract

The risk of anterior cruciate ligament (ACL) injury and re-injury is greater for women than men. Among other factors, compositional differences may play a role in this differential risk. Patellar tendon (PT) autografts are commonly used during reconstruction. The aim of the study was to compare protein expression in male and female ACL and PT. We hypothesized that there would be differences in key structural components between PT and ACL, and that components of the proteome critical for response to mechanical loading and response to injury would demonstrate significant differences between male and female. Two-dimensional liquid chromatography-tandem mass spectrometry and a label-free quantitative approach was used to identify proteomic differences between male and female PT and ACL. ACL contained less type I and more type III collagen than PT. There were tissue-specific differences in expression of proteoglycans, and ACL was enriched in elastin, tenascin C and X, cartilage oligomeric matrix protein, thrombospondin 4 and periostin. Between male and female donors, alcohol dehydrogenase 1B and complement component 9 were enriched in female compared to male. Myocilin was the major protein enriched in males compared to females. Important compositional differences between PT and ACL were identified, and we identified differences in pathways related to extracellular matrix regulation, complement, apoptosis, metabolism of advanced glycation end-products and response to mechanical loading between males and females. Identification of proteomic differences between male and female PT and ACL has identified novel pathways which may lead to improved understanding of differential ACL injury and re-injury risk between males and females.

## Introduction

There are over 80,000 anterior cruciate ligament (ACL) tears in the USA each year [1]. Anterior cruciate ligament (ACL) injury incidence is nearly 10 times higher in women than men engaged in similar activities [2], [3]. Female athletes are also more likely to present for revision or contra-lateral ACL reconstruction than their male counterparts [4]. Intrinsic and extrinsic risk factors for ACL injury in both males and females have been identified (reviewed in [2], [5]–[7]). The central third of the patellar tendon (PT) is commonly used as an autograft for ACL reconstruction. Therefore, differences in gene expression, ultrastructure and biochemical structure of male and female ACL and PT have been well studied [8]–[15]. However, we are not aware of previous studies using label-free protein quantitation to evaluate differential expression of proteins between male and female PT and ACL.

Potential advantages of a proteomic rather than conventional biochemical and antibody-based approaches include the ability to detect small differences in protein levels between samples and increased sensitivity and accuracy in protein identification. Furthermore, in other tissues correlation of mRNA and protein levels may be poor and depends partly on the abundance of the proteins involved and on complex post-transcriptional regulation and post-transcriptional modification pathways [16], [17]. Previously, proteomic techniques including two-dimensional gel electrophoresis (2D SDS-PAGE) have been used to evaluate the response of tendon and ligament to various stimuli [18]–[22]. However, potential disadvantages of 2D SDS-PAGE for evaluation of these tissues include the presence of highly abundant insoluble proteins such as mature type I collagen and the presence of anionic proteoglycans [23]. Shotgun proteomics involves the digestion of complex mixtures of proteins to peptides, evaluation by mass spectrometry, and annotation to identify proteins present. Label-free protein quantitation using two dimensional liquid chromatography-tandem mass spectrometry is used in complex samples to identify differences in protein abundance between samples [17], and may therefore be useful for evaluation of tendon and ligament.

The aim of this study was to compare the proteome of male and female PT and ACL using label-free protein quantitation. We hypothesized that there would be greater differences in key structural components between PT and ACL than between male and female donors, but that components of the proteome critical for collagen fibril organization, response to mechanical loading and response to injury would differ between male and female donors. These data provide new insight into pathways which may be involved in ACL injury, and potentially in the differential injury response of female ACL.

## Materials and Methods

### 2.1 Donors and Dissection

One intact knee joint from each of three male (Age 68–69 yrs, Body Mass Index (BMI, kg/m^2^) 31–33) and three female (Age 53–62 yrs, BMI 18–34) Caucasian donors were evaluated. The Duke University School of Medicine Anatomical Gifts Program provided the tissue samples (http://medschool.duke.edu/education/anatomical-gifts-program). Joints were frozen at −20°C within 12 hours of death. Donors had no previous history of knee trauma, surgery or arthritis, heterotopic ossification, diabetes, morbid obesity (BMI≥35) and were ambulatory immediately prior to death. All donors reportedly had moderate tobacco and alcohol use. Joints were thawed at 4°C, and the ACL and central third of the PT were dissected from their osseous and peritendinous attachments. Samples were rinsed with sterile 50 mM ammonium bicarbonate and stored at −80°C until analysis.

### 2.2 Protein Extraction

Samples were thawed, minced, rinsed further in sterile 50 mM ammonium bicarbonate, and lyophilized. Individual PT and ACL samples were pulverized in a freezer mill (Spex SamplePrep Freezer Mill 6770, SPEX SamplePrep, Metuchen, NJ), then stored at −80°C. Equal aliquots (13.7±0.1 mg dry weight) of each sample were washed in 1 mL (approximately 10x volumes) of 0.1% Rapigest (Waters Corporation, Milford, MA) in 50 mM ammonium bicarbonate and heated to 80°C for 10 minutes while shaking vigorously. Nine hundred µL of supernatant was removed after centrifugation and an additional 250 µL of 0.1% Rapigest was added to the precipitate. Samples were sonicated with a probe sonicator for 3 bursts of 5 seconds each, heated at 60°C for 10 minutes, reduced in 10 mM dithiothreitol for 15 minutes at 80°C, alkylated in 20 mM iodoacetamide for 30 minutes in the dark at room temperature, and digested in-situ with an estimated 25∶1 ratio of protein:trypsin overnight at 37°C. After overnight digestion, virtually 100% solubilization of the material was observed. Samples were acidified to 1% v/v trifluoroacetic acid to hydrolyze the Rapigest surfactant, concentration-normalized using a micro-bicinchoninic acid (BCA) assay (Thermo Fisher Scientific Inc., Rockford, IL), and spiked with alcohol dehydrogenase from *Saccromyces cerevisiae* (ADH1_YEAST) Massprep Digestion Standard (Waters) at 25 fmol/µg as a surrogate standard. Equal quantities of each sample were dried in a vacuum centrifuge and samples were resuspended in 100 mM ammonium formate at pH 10 before analysis.

### 2.3 Liquid Chromatography - Tandem Mass Spectrometry

Quantitative two-dimensional liquid chromatography – tandem mass spectrometry (LC/LC-MS/MS) was performed on 3 µg of protein digest per sample. Two-dimensional liquid chromatography in a high-low pH reversed phase/reversed phase configuration was used on a nanoAcquity ultra-performance liquid chromatography (UPLC) system (Waters) coupled to a Synapt G2 high definition mass spectrometer (HDMS) high resolution accurate mass tandem mass spectrometer (Waters) with nanoelectrospray ionization as described previously [24]–[26]. Peptides were first trapped at 2 µl/min at 97/3 v/v water/acetonitrile (MeCN) in 20 mM ammonium formate (pH 10) on a 5 µm XBridge BEH130 C18 300 um×50 mm column (Waters). Peptides were then eluted from the 1^st^ dimension column using a series of eight step-elutions of MeCN at 2 µL/min. Steps of 7.4%, 10.8%, 12.6%, 14.0%, 15.3%, 16.7%, 20.4% and 65.0% MeCN were used for the analyses; these percentages were selected specifically for the ACL and PT matrices based on a combination of total ion current and number of peptide identifications per fraction, for delivery of an approximately equal load to the 2^nd^ dimension column. For 2^nd^ dimension separation, the eluent from the 1^st^ dimension was first diluted 10-fold online with 99.8/0.1/0.1 v/v/v water/MeCN/formic acid and trapped on a 5 µm Symmetry C18 180 µm×20 mm trapping column (Waters). The 2^nd^ dimension separations were performed on a 1.7 µm Acquity BEH130 C18 75 µm×250 mm column (Waters). A linear gradient of 5 to 40% MeCN with 0.1% formic acid over 60 min was used at a flow rate of 0.4 µl/min and column temperature of 55°C. Quantitative data collection on the Synapt G2 mass spectrometer was performed in data-independent acquisition (MSE) mode, using 0.6 second alternating cycle time between low (6 V) and high (27–50 V) collision energy (CE). Scans performed at low CE measured peptide accurate mass and intensity (abundance), while scans at elevated CE allowed for qualitative identification of the resulting peptide fragments via database searching. Standard MSE acquisition was selected for quantitation since high-abundance ions have shown signal attenuation in the ion mobility mode also available on this instrumentation [27], and quantitation of high abundance collagen species was of particular importance to this study. Sample run order was randomized within sample type, and 5 out of the 6 samples per sample type were run in MSE mode one time while 1 of the 6 samples for each sample type was run in MSE mode in duplicate. The total analysis cycle time for each sample injection was approximately 11 hours.

Additionally, pools of each matrix type (either PT or ACL) were made by combining equivalent amounts of all 6 samples per type, and each was used to condition the UPLC column prior to the study. Each pool was also run in duplicate in data-dependent acquisition (DDA) mode to generate data files for supplementary identifications, and were also aligned with the quantitative (MSE) data (see Elucidator methods below) in order to translate any unique identifications from DDA to the corresponding peak in the quantitative datasets. DDA mode utilized a 0.6 sec MS scan followed by MS/MS acquisition on the top 3 ions with charge greater than 1. MS/MS scans for each ion used an isolation window of approximately 2.3 Da, 0.6 second scans with a maximum of 3 seconds per precursor, and dynamic exclusion for 120 seconds within 1.2 Da of the selected precursor m/z.

### 2.4 Data Alignment and Protein Identification

The data were collected and analyzed independently for ACL and PT. Within a tissue, data collection alternated between male and female subjects, to reduce any temporal bias. Including pooled sample analyses, replicate analysis for one sample of each matrix type, and 8 LC/LC fractions per sample, there were a total of 72 raw data files collected per matrix type.

Label-free quantitative analysis was performed independently for each tissue (PT and ACL) using area-under-the-curve (AUC) measurements in Rosetta Elucidator v3.3 (Rosetta Biosoftware Inc., Seattle, WA). Analyses for each LC/LC fraction were aligned based on the accurate mass and retention time of detected ions (“features”) using the PeakTeller algorithm (Elucidator), and after feature quantitation and identification, peptide quantities were summed across fractions for the small percentage of peptides found in multiple fractions (∼15%). MS/MS spectra generated via DDA were compiled and submitted to the Mascot v2.2 (Matrix Sciences Inc., London, UK, www.matrixscience.com) search engine directly from the Elucidator software (16,147 spectra for ACL and 17,963 for PT). For MSE data, ProteinLynx Global Server (PLGS) v2.4 (Waters) was used to generate searchable files, including 116,872 spectra for ACL and 130,385 for PT. These spectra were submitted to the IdentityE search engine within PLGS v2.4 on a fraction-by-fraction basis, with search parameters requiring 3 product ions per peptide, 7 product ions per protein, and 1 peptide per protein; results were then imported into Elucidator. All spectra were searched against a SwissProt (www.uniprot.org) database, including all *Homo sapiens* entries as well as for the surrogate standard ADH1_YEAST, which also contained a reversed-sequence “decoy” database for false positive rate determination. For Mascot and PLGS searches, data were searched for tryptic enzyme specificity, fixed modification for carbamidomethylation of cysteine residues, variable modifications of deamidation of asparagine and glutamine residues, hydroxylation of proline residues, and oxidation of methionine residues. Precursor ion tolerance was set at 10 ppm and 5 ppm and product ion tolerance was set at 0.04 Da and 12 ppm, for DDA and data independent acquisition (DIA) data respectively. A maximum of 2 missed cleavages were allowed. After individual peptide scoring using PeptideProphet algorithm (Elucidator), the data were annotated at a 2% peptide false discovery rate (FDR) for PT samples and 1.6% FDR at the peptide level for ACL. MS/MS identifications were made available as scaffold files and can be downloaded at the following link: https://discovery.genome.duke.edu/express/resources/2365/Little_MSMS_Supplement.7z.

### 2.5 Quantitative Data Analysis

To perform the most robust relative quantitation between male and female subjects within a tissue type, the intensities of all peptides per protein were summed for each subject [28] within the Rosetta Elucidator software package. The sum-protein intensities were averaged for the one sample which was analyzed twice for each tissue type. The fold-change difference in protein abundance between male and female subjects at the protein level was calculated from the ratio of average protein intensities for male and female. The statistical significance of this difference was calculated using an error-weighted ANOVA after log2 transform, with p-values reported after Benjamini-Hochberg FDR correction. Additional validation of the fold-change calculation between male and female subjects at the protein level within each tissue was performed by calculation of the effect size via Cohen's d. All proteins with a p-value <0.05 had an effect-size (Cohen's d) >0.8, which classified as a “large” effect size, thus the sample size of n = 3 for each sex and tissue type was adequate at the selected levels of statistical confidence (0.95) and significance (p<0.05) [29].

The proteomes of ACL and PT differed significantly enough to make Elucidator alignment of the datasets and direct comparison between tissues not feasible. Therefore the average intensity of the top 3 “best flier” peptides was used to obtain an estimate of mol (or ng) quantity of protein in each sample as has been described previously [30], [31]. The ‘best flier’ peptide method is a high-throughput method to estimate protein quantity and is accurate to within approximately a two-fold change compared to highly orthogonal methods [32]. Based on the average intensity of the top 3 peptides for the surrogate standard of known concentration in each sample (ADH1_YEAST), the weight was estimated for all proteins with at least two peptides in ACL or PT. The weight of each protein was then normalized to the total protein in each sample to express each protein as a fraction of the dry weight of the whole, similar to previously described [33]. For proteins which were present in both ACL and PT at quantifiable levels, the fold-changes between PT and ACL were calculated globally across all samples (n = 6), and the statistical confidence of these changes was estimated using Students T-test and Cohen's d as described above. Fold-changes and T-test p-values were also reported for male and female comparisons of ACL to PT independently.

Tendon and ligament are known to contain a wide variety of ‘matrisomal proteins’ [34]. The matrisome is the fraction of the proteome that represents the full complement of extracellular matrix proteins [34]. Matrisomal proteins include members of the collagen superfamily, proteoglycans including the small leucine-rich proteoglycans (SLRP family), glycoproteins and proteins otherwise associated with the ECM. Therefore, the Matrisome Project was used to annotate matrisomal proteins identified in this study to extracellular matrix categories [34]–[36]. Tendon and ligament are also vascular tissues, but proteins present in blood may also be differentially expressed between tissues and sexes. Therefore we interrogated the proteomic datasets for these proteins. In order to identify proteins present in blood, gene ontology terms associated with blood were applied to protein lists uploaded into The Database for Annotation, Visualization and Integrated Discovery (DAVID, National Institute of Allergy and Infectious Diseases, NIH) [37], [38]. Candidate proteins were then evaluated for possible enrichment in ACL and PT compared to the intravascular protein albumin. In addition, several proteins are known to play a role in tendinopathy [39]–[43], therefore the datasets were additionally evaluated for presence and enrichment of these known proteins.

## Results

Peptide-level quantitative data for each sample, along with identification scores for each search engine, peptide mass, charge, and retention time are available as two supplementary data files (ACL in **Table S1** and PT in **Table S2**). Results of summed intensities of all peptides for each protein for each subject and differential abundance data are available in **Table S3** (ACL) and **Table S4** (PT). The quantitative values for each protein, in both ACL and PT for each sample are reported in **Table S5**. For proteins which were present in both ACL and PT at quantifiable levels, the fold-changes between both PT and ACL and male and female within these tissues and the statistical confidence of these changes are also reported in **Table S5**.

### 3.1 2DLC-MS/MS Data Quality Control

**Figure 1:**
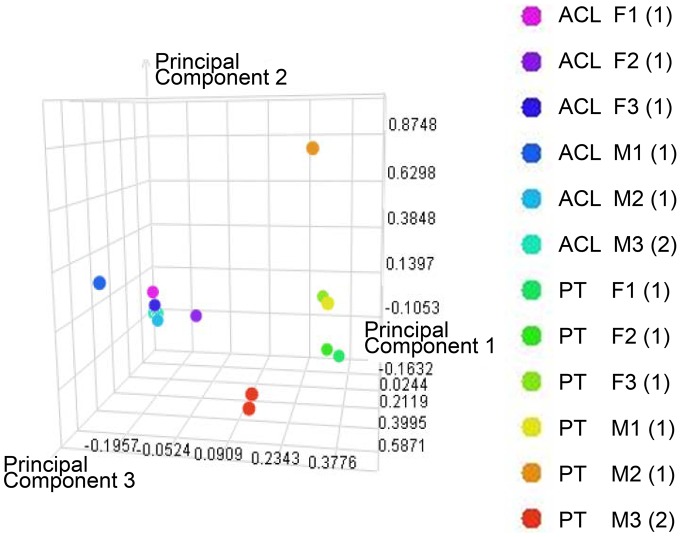
Principal components analysis (PCA) using the peptide-level quantitative data from Tables S1 and S2. PCA after z-score normalization and calculation of the top 3 principal components. ACL, anterior cruciate ligament; PT, patellar tendon; M1–3, male donor 1–3; F1–3, female donor 1–3. Number in brackets represents number of technical replicates performed.

The relative standard deviation (RSD) was calculated at the protein-level from **Tables S3** and **S4** for the single protein surrogate standard (ADH1_YEAST) that was spiked into each sample to estimate the precision of the label-free protein quantitation across all samples. RSD for the surrogate standard was found to be 5% for the ACL dataset and 21% for the PT dataset, both respectable values. Principal components analysis (PCA) is a data reduction method which assists in visualization of relative variability between biological samples and technical repeats; PCA was performed in the Rosetta Elucidator software package using the peptide-level quantitative data from **Tables S1** and **S2** after z-score normalization, and the top 3 principal components calculated were plotted in three-space (**Figure 1**). ACL and PT were well separated along principal component (PC) 1, but within each tissue the male and female samples occupied very similar space in PC 2 and 3. The single sample of each tissue that was analyzed in duplicate (annotated ACLM3 and PTM3) overlaid almost exactly on top of the other, suggesting that the biological variability was much larger than the variability contributed by the analysis platform.

The ability of the high/low pH 2DLC method to evenly and reproducibly fractionate the tissues across the two-dimensional separations space was also analyzed. From **Table S1** and **S2**, the percentage of peptides in each sample type that were found in a single fraction was calculated. The reversed-phase/reversed-phase 2DLC separation provided a very high degree of fraction uniqueness, with 82% and 83% of the peptides in a single LC fraction for ACL and PT analyses, respectively. On evaluation of the number of peptides and the total ion current in each 2DLC fraction, the column load was relatively evenly distributed across the fractions for both ACL and PT (**Figure S1**), although higher total ion current (TIC) and lower peptide counts were observed for the early fractions, presumably due to the relatively high quantity of hydroxyproline domain peptides from collagen isoforms.

### 3.2 Qualitative Interrogation of the ACL and PT Proteomes

We identified 1586 and 2022 peptides assigned to 178 and 166 native proteins in ACL and PT respectively (**Table S3** and **S4**). Of the 178 proteins identified in ACL, 83 (47%) were identified with the presence of more than 2 peptides (**Table S5**). Similarly, 68 of 166 (41%) of the proteins identified in PT were identified with more than 2 peptides (**Table S5**). Of these, 86 proteins were identified in both ACL and PT. Based on annotation from the Matrisome Project [34], [36], in ACL, there were 14 proteins annotated as collagens, 12 proteoglycans, 23 glycoproteins, 11 ECM-regulators, 5 ECM-affiliated, and 1 ECM-secreted factor. In PT, there were 13 proteins annotated as collagens, 8 proteoglycans, 24 glycoproteins, 9 ECM-regulators, 6 ECM-affiliated, and 3 ECM-secreted factors. False discovery rate (FDR) for ACL and PT was 1.6% and 2% respectively.

### 3.3 Quantitative Differences between Male and Female Tissues

**Table 1** was curated from **Table S3** and **S4**, by requiring >1 peptide to match, an ANOVA p-value <0.05 after Hochberg FDR correction, and Cohen's d for the individual analyte of >0.8, a “large” effect size, then fold-changes and T-test p-values were reported for male and female comparisons of proteins meeting these criteria for ACL and PT independently.

**Table 1:** Differentially expressed proteins between male and female in either anterior cruciate ligament (ACL), or patellar tendon (PT) or both.

			ACL				PT			
Primary Protein Name	Protein Description	GENE	Peptide Count	Fold Change M/F	ANOVA p-value	Cohen's d	Peptide Count	Fold Change M/F	ANOVA p-value	Cohen's d
ACTH_HUMAN†	Actin, gamma-enteric smooth muscle	ACTG2	*8*	*−1.14*	*8.29E-01*	*−0.27*	9	1.76	1.80E-02	2.08
ADH1B_HUMAN*	Alcohol dehydrogenase 1B	ADH1B	2	−4.98	2.00E-03	−3.54	*1*	*−8.64*	*9.38E-15*	*−9.59*
ALBU_HUMAN	Serum albumin	ALB	40	2.24	1.41E-08	5.40	62	2.48	6.25E-14	8.10
ANXA2_HUMAN	Annexin A2	ANXA2	*3*	*1.38*	*8.25E-01*	*0.74*	6	1.56	1.80E-02	2.13
CO1A1_HUMAN	Collagen alpha-1(I) chain	COL1A1	210	−1.20	8.83E-04	−4.29	520	−1.20	2.10E-02	−2.12
CO1A2_HUMAN	Collagen alpha-2(I) chain	COL1A2	186	−1.23	7.00E-03	−3.06	374	−1.12	4.20E-02	−1.76
CO6A1_HUMAN	Collagen alpha-1(VI) chain	COL6A1	*50*	*−1.00*	*8.99E-01*	*−0.02*	61	1.41	4.50E-02	2.29
CO9_HUMAN	Complement component C9	C9	3	−4.56	1.15E-09	−3.94	*2*	*−2.10*	*2.44E-01*	*−1.50*
LUM_HUMAN	Lumican	LUM	*13*	*−1.02*	*8.27E-01*	*−0.08*	9	1.81	9.38E-15	10.36
MIME_HUMAN	Mimecan	OGN	*15*	*1.30*	*8.30E-01*	*0.55*	7	1.31	1.99E-06	5.15
MMP3_HUMAN	Stromelysin-1	MMP3					4	−2.14	7.17E-04	−2.27
MYOC_HUMAN	Myocilin	MYOC	3	3.88	2.00E-03	2.57	4	1.20	8.53E-01	0.38
PGS2_HUMAN	Decorin	DCN	26	−1.22	9.45E-09	−6.42	25	−1.02	7.51E-01	−0.36
TSP1_HUMAN	Thrombospondin-1	THBS1	*3*	*−1.32*	*8.27E-01*	*−0.83*	5	−2.04	1.80E-02	−1.95
TTHY_HUMAN	Transthyretin	TTR	*14*	*21.72*	*6.72E-01*	*0.98*	2	1.83	2.30E-02	1.87
VIME_HUMAN	Vimentin	VIM	*6*	*1.66*	*3.24E-01*	*1.18*	3	2.01	1.90E-02	1.99

The table was curated from Tables S3 and S4 using >1 peptide count for either ACL or PT, an ANOVA p-value <0.05 after Hochberg FDR correction for ACL or PT, and Cohen's d for the individual analyte of >0.8. If these criteria were not met for both ACL and PT, the data for the other tissue (italics) were included for completeness.

*A single peptide was identified in PT which was homologous to ADH1B, ADH1C, and ADH1G. This single peptide was annotated somewhat arbitrarily to ADH1G in the PT dataset (Table S2) because of ProteinProphet homology rules and was also identified and quantified in the ACL dataset (Table S1). The statistical data has been inserted here for ADH1B because of its consistency between ACL and PT.

† all peptides identified as ACTS in PT also map to ACTH in ACL, but the converse is not true for all peptides mapped as ACTH in ACL. Therefore both identifications were represented as ACTH.

Seven proteins were differentially expressed in ACL between male and female, and 12 proteins were differentially expressed in PT between male and female (**Table 1**). Alcohol dehydrogenase 1B (ADH1B) was most enriched in female compared to male, followed by complement component 9 (CO9), stromelysin-1 (MMP3) and thrombospondin-1 (TSP1). Myocilin (MYOC), serum albumin (ALBU) and vimentin (VIME) were most highly enriched in male compared to female.

### 3.4 Quantitative Differences between PT and ACL

**Table 2** shows a subset of 29 proteins from **Table S5** which was curated for differential expression between ACL and PT using T-test p<0.05 and a Cohen's d>0.8 for each analyte. Fibronectin (FINC), complement component C9 (CO9) and histidine-rich glycoprotein (HRG) were the most enriched proteins of 23 proteins enriched in ACL compared to PT (**Table 2**). Type XII collagen (COL12A1), alpha-1-microglobulin/bikunin precursor (AMBP) and fibromodulin (FMOD) were the most enriched proteins of 6 proteins enriched in PT compared to ACL (**Table 2**).

**Table 2:** Differentially expressed proteins between anterior cruciate ligament (ACL) and patellar tendon (PT).

PrimaryProtein Name	Protein Description	Gene Name	ACL Peptide Count	PT Peptide Count	Fold Change ACL/PT	p-value ACL/PT	Cohen's d	Female Fold Change ACL/PT	Female p-value	Male Fold Change ACL/PT	Male p-value
FINC_HUMAN	Fibronectin	FN1	72	32	6.00	6.78E-04	2.17	7.84	2.50E-02	4.97	4.55E-02
CO9_HUMAN	Complement component C9	C9	3	2	5.64	2.18E-02	1.22	6.97	1.12E-02	3.21	3.87E-02
HRG_HUMAN	Histidine-rich glycoprotein	HRG	2	2	4.74	1.78E-03	1.91	6.94	7.63E-02	3.62	2.50E-02
MIME_HUMAN	Mimecan	OGN	15	7	4.44	2.53E-02	1.13	4.51	2.81E-02	4.40	1.88E-01
CO3A1_HUMAN	Collagen alpha-1(III)	COL3A1	135	90	4.10	5.74E-08	7.29	5.50	3.61E-05	3.19	1.16E-03
VIME_HUMAN	Vimentin	VIM	6	3	3.53	4.43E-02	1.00	3.93	3.40E-03	3.37	1.75E-01
CLUS_HUMAN	Clusterin	CLU	16	12	3.53	4.86E-04	2.42	3.29	1.23E-03	3.87	7.62E-02
TENX_HUMAN	Tenascin-X	TNXB	28	9	3.43	3.34E-03	1.69	3.52	6.46E-02	3.38	9.61E-03
ASPN_HUMAN	Asporin	ASPN	17	9	3.27	4.49E-03	1.78	3.27	3.93E-03	3.28	1.16E-01
BGH3_HUMAN	Transforming growth factor-beta-induced protein ig-h3	TGFBI	10	11	3.11	1.57E-03	2.06	3.32	3.66E-02	2.97	5.07E-02
PGS1_HUMAN	Biglycan	BGN	22	18	3.06	1.66E-04	2.59	3.05	2.38E-02	3.08	5.88E-03
ANXA1_HUMAN	Annexin A1	ANXA1	4	2	3.05	7.29E-05	3.86	4.11	6.71E-03	2.51	6.76E-03
ANXA5_HUMAN	Annexin A5	ANXA5	5	4	3.02	6.66E-04	2.54	3.53	2.68E-02	2.72	2.20E-02
TENA_HUMAN	Tenascin C	TNC	6	3	2.99	1.43E-02	1.29	2.49	1.41E-01	3.33	3.98E-02
ELN_HUMAN	Elastin	ELN	5	4	2.53	2.65E-03	1.89	3.04	6.99E-04	2.17	1.74E-01
TSP4_HUMAN	Thrombospondin-4	THBS4	10	15	2.46	3.93E-03	1.69	2.22	1.12E-01	2.63	2.36E-03
PRELP_HUMAN	Prolargin	PRELP	40	29	2.46	4.67E-04	2.26	2.52	1.35E-02	2.40	3.68E-02
CILP1_HUMAN	Cartilage intermediate layer protein 1	CILP	62	39	2.42	4.91E-04	2.65	2.37	5.72E-02	2.45	7.54E-03
PGBM_HUMAN	Basement membrane-specific heparan sulfate proteoglycan core protein	HSPG2	6	4	2.32	9.52E-04	2.18	2.43	9.37E-03	2.24	4.65E-02
H4_HUMAN	Histone H4	HIST1H4A	3	7	2.25	2.75E-02	1.16	2.25	1.40E-01	2.25	1.77E-01
LUM_HUMAN	Lumican	LUM	13	9	2.07	3.22E-02	1.16	2.92	9.27E-05	1.69	3.14E-01
A1AT_HUMAN	Alpha-1-antitrypsin	SERPINA1	3	4	1.72	3.16E-02	1.58	1.61	1.87E-01	1.80	1.29E-01
COMP_HUMAN	Cartilage oligomeric matrix protein	COMP	48	51	1.63	2.66E-02	1.33	1.54	2.59E-01	1.71	3.01E-02
CO1A2_HUMAN	Collagen alpha-2(I)	COL1A2	183	374	−1.51	1.47E-04	−4.83	−1.58	1.24E-03	−1.43	5.58E-02
CO1A1_HUMAN	Collagen alpha-1(I)	COL1A1	200	520	−1.60	3.68E-08	−11.94	−1.58	7.94E-04	−1.62	4.60E-04
FBN1_HUMAN	Fibrillin-1	FBN1	34	58	−1.62	1.22E-02	−1.80	−1.63	1.26E-02	−1.61	1.74E-01
FMOD_HUMAN	Fibromodulin	FMOD	6	15	−2.92	6.50E-04	−4.34	−3.45	3.08E-02	−2.44	1.69E-02
AMBP_HUMAN	Protein AMBP	AMBP	7	10	−3.92	4.24E-04	−3.07	−2.77	6.82E-02	−6.54	7.71E-03
COCA1_HUMAN	Collagen alpha-1(XII)	COL12A1	8	69	−6.22	2.45E-06	−9.01	−7.87	8.82E-04	−5.27	4.62E-03

The table was curated from Table S5 using >1 peptide count for either ACL or PT, an ANOVA p-value <0.05 after Hochberg FDR correction for ACL or PT, and Cohen's d for the individual analyte of >0.8. This table only includes those proteins present and quantified in both tissue types. Differential expression between ACL and PT for female and male is also identified, with their respective p-values.

### 3.5 Matrisomal Protein Distributions

Figures for matrisomal protein families represented in ACL and PT were produced from quantitative data in **Table S5**.

#### 3.5.1Collagen Superfamily

**Figure 2:**
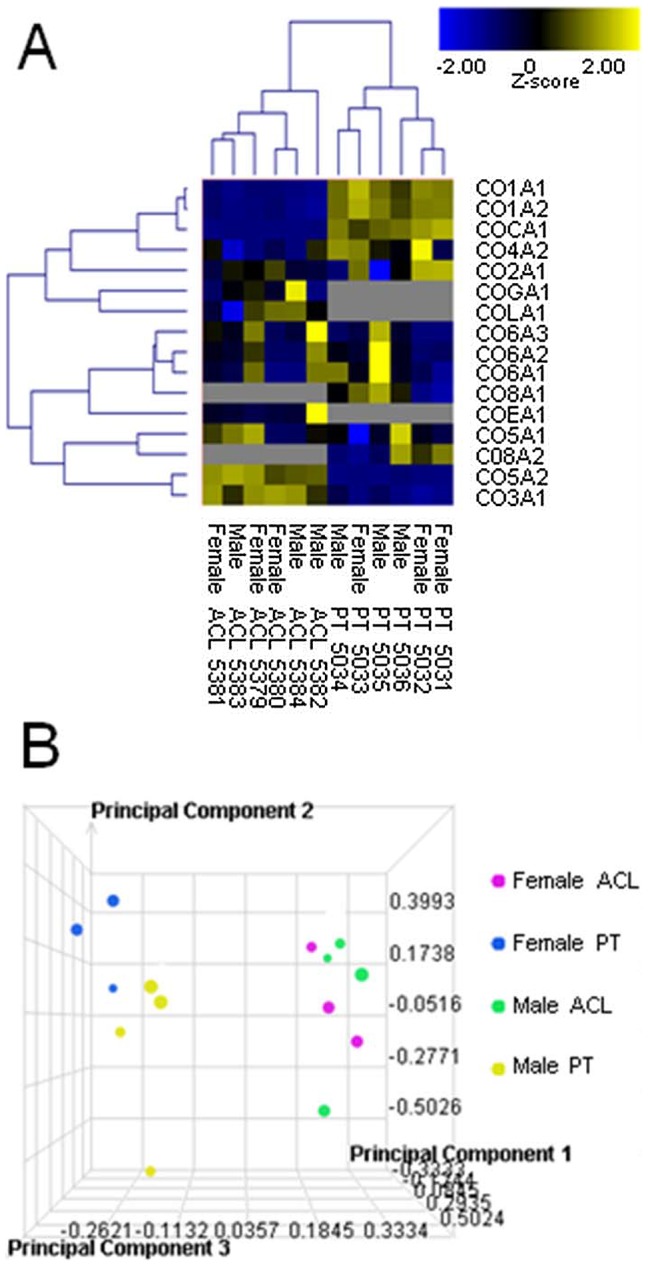
Agglomorative clustering analysis (A) and Principal Components Analysis (B) of members of the collagen superfamily. Members of the collagen superfamily identified in males and female anterior cruciate ligament (ACL) and patellar tendon (PT).

Sixteen chains of the collagen superfamily, representing 11 members, were identified. Types XIV (COEA1), XVI (COGA1) and XXI (COLA1) collagen were identified in ACL but not in PT (**Figure 2a**), and type VIII (CO8A1 and CO8A2) collagen was identified in PT but not ACL. PCA (**Figure 2b**) using these 16 collagen chains demonstrated good separation between ACL and PT, but differences between male and female were less obvious.

**Figure 3:**
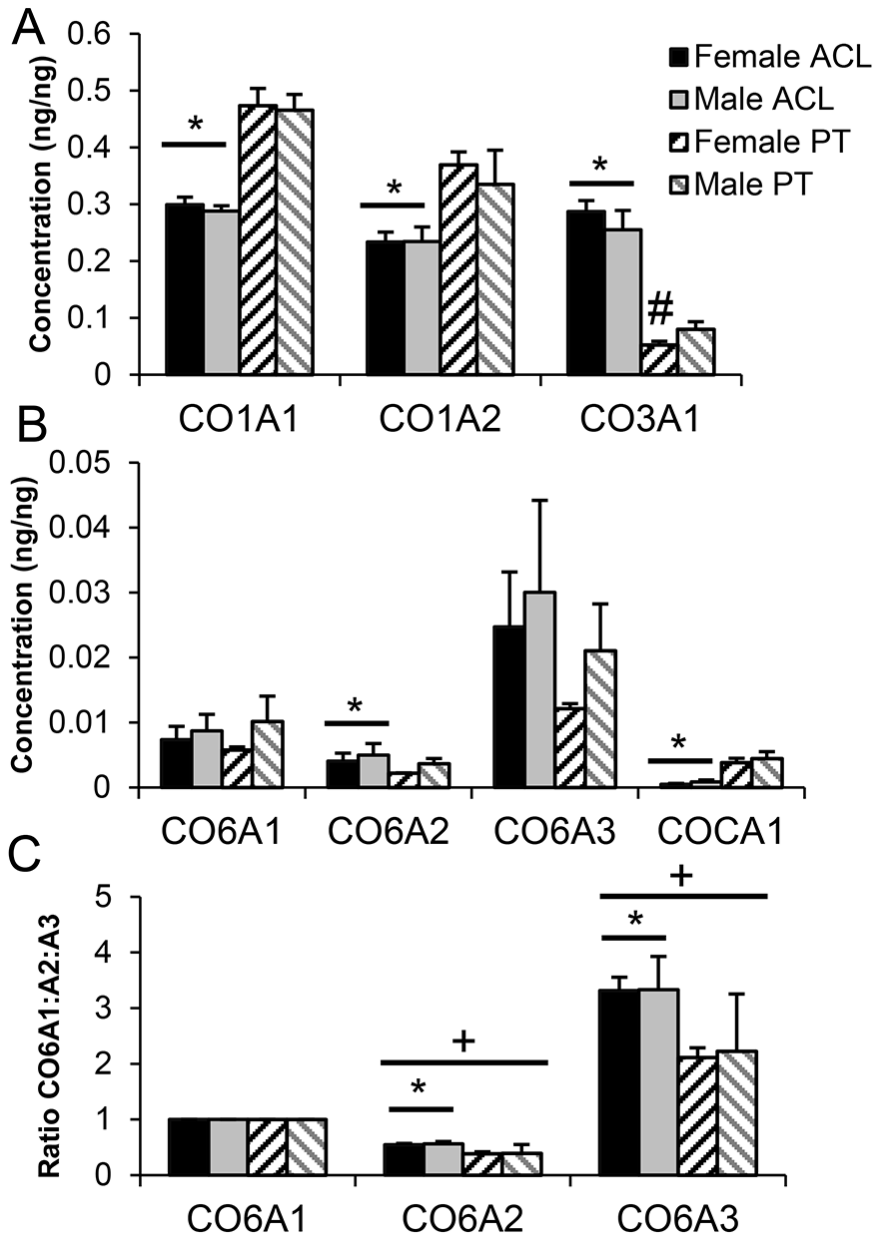
Concentrations (A,B) and ratio of CO6A1:CO6A2:CO6A3 (C) of members of the collagen superfamily. * Patellar tendon (PT) significantly different to anterior cruciate ligament (ACL), # female different to male within tissue, + significantly different to CO6A1:CO6A1. (Mean ± SD).

Type I collagen was marginally enriched in female compared to male (**Table 1**), but this was not identified as significant after comparison of the results of direct quantification was made (**Figure 3**). However, enrichment of type III(α1) collagen (CO3A1) in ACL and type I collagen (CO1A1 and CO1A2) in PT (**Table 1**) was confirmed (**Figure 3**). After direct quantitation, male PT had significantly more CO3A1 than female PT (p = 0.04, **Figure 3**). Total (CO1A1+CO1A2) type I collagen (% dry weight) of PT was significantly greater than ACL (82.16%±3.34 compared to 52.74%±1.25, p = 0.000001), and type III collagen content of PT was significantly less than ACL (6.61%±1.79 compared to 27.11%±3.04, p<0.0001), which gave a significantly greater type I∶III collagen ratio for PT compared to ACL (1∶13.28±3.86 compared to 1∶1.97±0.24, p = 0.001).

Type II(α1) collagen (CO2A1) was expressed in both tissue types, but levels were not different between ACL and PT, or between sexes. Type V(α2) collagen (COL5A2) was identified in ACL but not PT, and was not differentially expressed between male and female. Type VI(α1) collagen (COL6A1) was enriched in male PT compared to female PT (**Table 1**), but this difference was not identified as significant after comparison of the results of direct quantification (**Figure 3B**). Type VI(α2) collagen (COL6A2) was increased in ACL compared to PT, but was not differentially expressed between male and female. After direct quantification, the ratio of COL6A1:COL6A2:COL6A3 was significantly different from the expected 1∶1∶1, therefore the ratios for individual donors and tissues were calculated (**Figure 3C**). Ratio of CO6A2:COL6A1 (p = 0.02) and COL6A3:COL6A1 (p = 0.02) was significantly greater in ACL than PT, but there was no effect of sex.

#### 3.5.2 Proteoglycans

**Figure 4:**
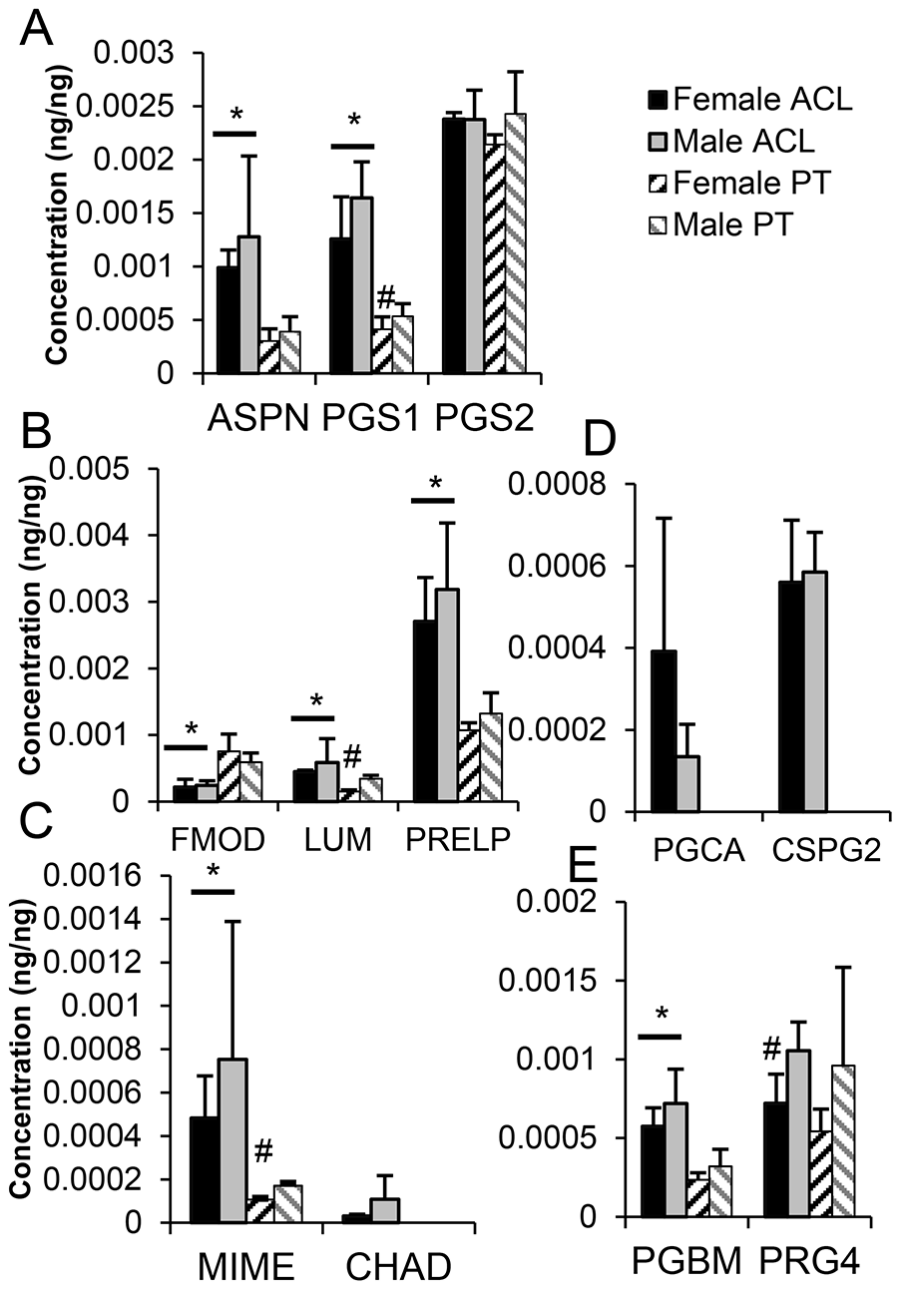
Concentrations of members of Class I (A), II (B) and III and IV(C) of the small leucine-rich repeat proteoglycans, the hyalectans (D), and other proteoglycans (E). * Patellar Tendon (PT) significantly different to anterior cruciate ligament (ACL), # female different to male within tissue.

Twelve proteoglycans were identified in ACL and of these, 9 were also identified in PT. Eight members of four classes of SLRPs were identified (**Figure 4A–C**). Aggrecan (PGCA) and versican (CSPG2) were identified in ACL but not PT (**Figure 4D**). With the exception of fibromodulin (FMOD), which was enriched in PT compared to ACL, decorin (PGS2) and lubricin (PRG4) (**Figure 4E**) where no difference was found between ACL and PT, all other proteoglycans were enriched in ACL compared to PT. Lumican (LUM), biglycan (PGS1), mimecan (MIME) and lubricin (PRG4) demonstrated enrichment in male donors. PGS2 demonstrated marginal but significant enrichment in female (**Table 1**), which was not confirmed after direct quantitation (**Figure 4A**).

#### 3.5.3 Glycoproteins

Twenty-three glycoproteins were identified in ACL, and 22 in PT, of which 17 were common to both ACL and PT. Of these, 7 were associated with microfibrils and elastic fibers [44], both known components of tendon and ligament. No differences between male and female were identified in these glycoproteins. Only elastin (ELN) and fibrillin-1 (FBN-1) were differentially enriched between ACL and PT (**Table 2**); ELN was enriched in ACL compared to PT, and FBN1 was enriched in PT compared to ACL. Of the other glycoproteins identified, cartilage intermediate layer protein-1 (CILP) and cartilage oligomeric matrix protein (COMP) were enriched in ACL compared to PT (**Table 2**), and tenascin-X (TENX), tenascin-C (TENA) and thrombospondin-4 (TSP4) were enriched in ACL compared to PT (**Table 2**). Thrombospondin-1 (TSP1) was the only glycoprotein differentially expressed between male and female and this was enriched in female PT (**Table 1**). However, this difference was not identified as significant after comparison of the results of direct quantification (**Table S5**).

#### 3.5.4 ECM-regulatory proteins

**Figure 5:**
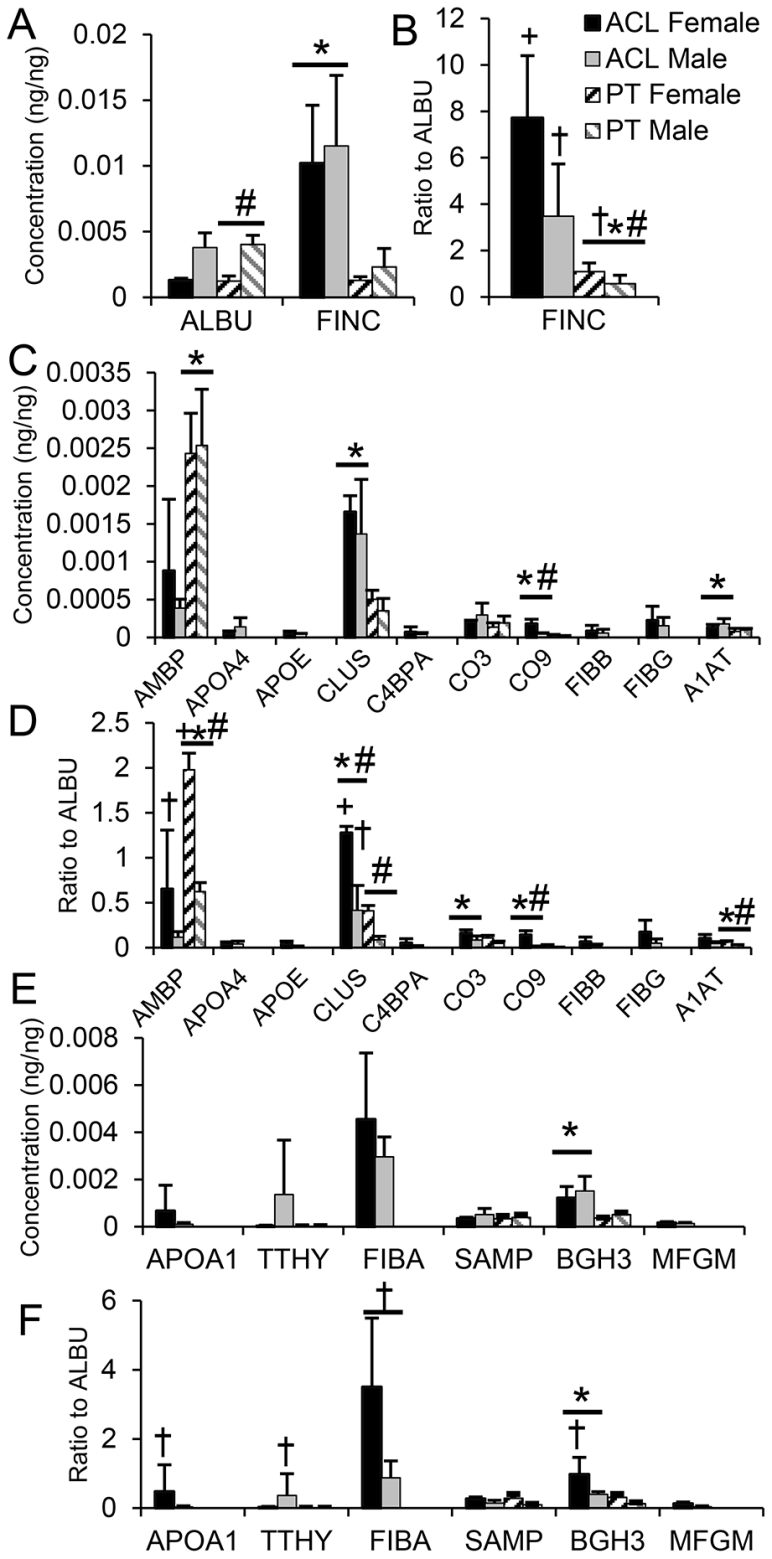
Concentrations (A,C,E), and ratio of protein to albumin (ALBU) (B,D,F) of plasma proteins (A,B,C,D) and amyloid proteins (E,F). * Patellar Tendon (PT) significantly different to anterior cruciate ligament (ACL), # female different to male, + significantly greater than ALBU:ALBU, † Not significantly different from ALBU:ALBU. Statistical comparisons were only made between ACL and PT if the protein was identified in both tissues. (Mean ± SD).

Thirteen proteins were identified with known ECM-regulatory function. Of known relevance to tendon and ligament, matrix metalloproteinase (MMP3) was only identified in PT and was enriched in female compared to male (**Table 1**). MMP10 and MMP11 were only identified in ACL. Tissue inhibitor of metalloproteinase 3 (TIMP3) was identified in both ACL and PT. A disintegrin and metalloproteinase 15 (ADA15) was identified in PT but not ACL, and a disintegrin and metalloproteinase with thrombospondin motifs 7 (ATS7) was identified in ACL but not PT. Of the other proteins identified with known ECM-regulatory function, histidine-rich glycoprotein (HRG) and alpha-1-antitrypsin (A1AT) were enriched in ACL (**Table 2**), and alpha-1-microglobulin/bikunin precursor (AMBP) was enriched in PT (**Table 2** **,** **Figure 5**).

### 3.6 Proteins Related to Tendon or Ligament Disease

Several proteins identified have previously been associated with tendinopathy or are known to be involved in proposed mechanisms of tendinopathy, including hypoxia or apoptosis. Annexin A1 (ANXA1) and A5 (ANXA5) were enriched in ACL (**Table 2**), and Annexin A2 (ANXA2) was enriched in male PT compared to female (**Table 1**). S100-A10 (S10AA), the binding partner of ANXA2 was identified in PT, but not in ACL (**Table S4**). Periostin (POSTN) was identified in both ACL and PT. Complement component 9 (CO9) was highly enriched in ACL compared to PT and in female compared to male (**Tables 1** **&** **2** **,** **Figure 5**). Clusterin (CLUS) was enriched in ACL compared to PT, and was significantly enriched compared to albumin in female ACL (**Figure 5**).

### 3.7 Blood, Serum and Amyloid Proteins

As a vascular tissue, proteins from blood bathe tendon or ligament extracellular matrix in intravascular and extravascular interstitial fluid, but are not typically interrogated as part of tendon or ligament extracellular matrix. Nonetheless, they may play a role in tendon and ligament homeostasis and disease and may be enriched in different tendon and ligament structures. To investigate this possibility, in the context of proteomic differences between male and female ACL and PT for proteins identified in Table S5 and annotated by the Gene Ontology terms ‘blood, plasma, immunoglobin, amyloid, amyloidosis, hemostasis’ in DAVID, both the quantitative differences between the two tissue types for male and female were evaluated (**Figure 5A, C, E**), in addition to their relationship to albumin (ALBU), a highly abundant blood protein (**Figure 5B, D, F**). Albumin (ALBU) is present in serum at concentrations of 20–50 mg/mL [45], and is one of the most abundant blood proteins, thus as a conservative estimate any blood, serum or amyloid protein present in a vascularized tissue at similar or higher levels than albumin would be expected to be preferentially enriched within the tissue. For example, in line with previous studies [8], fibronectin (FINC)was enriched compared to ALBU and in ACL compared to PT. Alpha-1-antitrypsin (A1AT), transforming growth factor-beta-induced protein Ig-h3 (BGH3), complement component 9 (CO9) and clusterin (CLUS) were enriched in ACL compared to PT, but alpha-1-microglobulin/bikunin precursor (AMBP) was enriched in PT. After normalization to ALBU, AMBP demonstrated relative enrichment in female PT compared to male PT and CLUS demonstrated relative enrichment in female ACL and PT relative to male ACL and PT. CO9 demonstrated relative enrichment in female compared to male ACL (**Figure 5C**).

## Discussion

### 4.1 Summary of Major Findings

Significant quantitative differences were identified between the proteome of male and female ACL and PT. ACL contained less type I collagen and more type III collagen than PT, and in both tissues, the ratio of collagen α1(VI):α2(VI):α3(VI) monomers was significantly different from the anticipated 1∶1∶1. There were specific differences in expression of proteoglycans between tissues. ACL contained more elastin, tenascin X, tenascin C, COMP, thrombospondin 4 and periostin than PT. Compared to ACL, alpha-1-microglobulin/bikunin precursor was enriched in PT at similar levels to albumin.

Between male and female donors, alcohol dehydrogenase 1B was the protein most enriched in female compared to male, followed by complement component 9. Thrombospondin 1 and clusterin were also enriched in females compared to males. Conversely, myocilin was the major protein enriched in males compared to females.

These findings support the hypotheses that there would be greater differences in key structural components between PT and ACL tissues than between male and female donors, but that components of the proteome critical for collagen fibril organization, response to mechanical loading and response to injury would differ between male and female tissues.

### 4.2 Evaluation of Results Compared to Previously Reported Studies

Comparison of the differences between ACL and PT revealed many similarities to previously reported studies (**Table 3**). Where differences were noted, this may have been due in part to poor correlation between mRNA expression and protein levels, or to differences in species or age of donor. Poor correlation between mRNA expression and protein levels may be dependent on the abundance of the specific proteins, and on the presence of complex post-transcriptional regulation pathways [16], [17].

**Table 3:** Comparison of results of compositional or gene expression analysis from previous studies evaluating differences between ACL and PT or differences between male and female PT to quantitative results from the current study.

Comparison & Method	Gene/Protein Name	Result	Current Study	Reference
**Rabbit ACL vs PT**	Fibronectin	ACL>PT	+	[8]
*Protein ELISA*				
**Human ACL vs PT**	Type I Collagen	ACL<PT	+	[11]
*Immunohistochemistry*	Type III Collagen	ACL>PT	+	
	Type V Collagen	ACL>PT	+	
	Elastin	ACL>PT	+	
**Porcine ACL vs PT**	COL2A1	ACL<PT	− ACL = PT	[13]
*Gene Array* †	ACAN	ACL>PT	+	
	MYOC	ACL<PT	− ACL = PT	
	COMP	ACL>PT	+	
	FMOD	ACL<PT	+	
	TSP4	ACL<PT	+	
	TNC	ACL>PT	− ACL>PT	
**Human Male vs Female PT**	COL3A1	Female>Male	− Male>Female	[14]
*Gene Expression*	COL1A1	Female = Male	+/− Female>Male	
	DCN	Female = Male	+/− Female>Male	
	BGN	Female = Male	+/− Male>Female	
	FMOD	Female = Male	+	
	MMP3	Male>Female*	Female >Male*	

+ Similar finding or similar trend in current study to previous study. – Different finding or different trend in current study to previous study. +/− Consistent with previous work.

*Trend to significance (≤0.08).

† Statistical differences between individual normalized gene expression values not reported in original study.

### 4.3 Collagen Superfamily

Proportions of the two most abundant collagens in tendon and ligament (type I + III collagen) were similar to those reported for rabbit PT and cruciate ligaments [46]. In contrast, in this study, type II collagen was present at similar levels in ACL and PT and there was no effect of sex. Type II collagen gene expression has previously been reported to be greater in porcine PT than ACL [13]. Localization of type II collagen expression in fibrocartilage of the normal human ACL has been reported at the site of compression of the ACL against the intercondylar fossa during full knee extension [47]. Similarly, compressive strains have been measured in the PT as it passes over the inferior pole of the patellar [48], and corresponds to a region of fibrocartilage in some patients with patellar tendinopathy [49], [50]. Thus differences in type II collagen expression between human and pig may reflect differences in distribution of fibrocartilage associated differences in regions of compressive strains between ACL and PT during normal gait.

Type VI collagen is a triple-helical monomer typically found with equal mRNA expression of α1(VI), α2(VI) and α3(VI) subunits [51]. However, α4(VI), α5(VI) and α6(VI) subunits have recently been reported, suggesting that more complex macro-molecular assemblies may be possible [52], [53]. Further, increased α3(VI) gene expression has been described in adipose tissue in obesity [54], [55], and in a recent proteomic study, α2(VI) but not α3(VI) was up-regulated in omental adipose tissue from women with gestational diabetes mellitus [56]. The current study identifies a ratio for α1(VI):α2(VI):α3(VI) of approximately 2∶1∶6 for ACL and 2∶1∶4 for PT, suggesting type VI collagen subunits may be differentially regulated in these tissues, and that regulation of type VI collagen chains within individual tissues may be complex.

### 4.4 Proteoglycans

Identification of the hyalectans (aggrecan and versican) in ACL confirms findings of previous studies, where perifibrillar localization of aggrecan was found in canine ACL, and versican was identified in lapine ACL [57], [58]. The majority of the SLRPs were elevated in ACL compared to PT. Proline/arginine-rich end leucine-rich repeat protein (PRELP) was the predominant SLRP in ACL, followed by decorin, biglycan, asporin and lubricin. In contrast, in PT, decorin was the predominant species, followed by PRELP, lubricin, biglycan and asporin. These findings contrast to other studies, which have suggested that biglycan and decorin are the predominant SLRP species in tendon [59]. Whether or not the distribution of SLRPs in these donors is representative of the distribution in younger donors remains undetermined. The predominance of PRELP was interesting given previous conflicting reports of its presence in tendon [60]–[62], and recent evidence to suggest a critical role in ligament formation [63]. In addition, the pattern of expression of PRELP in PT and ACL was similar to that of complement component 9, suggesting that PRELP may inhibit complement in ACL and PT as occurs in synovial fluid [64]. The role of asporin is unknown, but it competes for type I collagen binding with decorin, but not biglycan [65], inhibits periodontal ligament mineralization [66], [67], and is expressed in degenerate intervertebral disc [68]. These data suggest that asporin may function to prevent mineralization in degenerate or aged tissues. Mimecan regulates collagen fibrillogenesis [69], and the expression of biglycan and CHAD [70]. Fibromodulin and lumican had a complementary enrichment pattern, as would be expected from their shared binding sites [23], [71]. Together these data suggest a highly complex relationship between proteoglycans in PT and ACL and suggest proteoglycans in tendon may function in cellular homeostasis and response to mechanical loading in addition to regulation of collagen fibrillogenesis.

### 4.5 Glycoproteins

There was no consistent pattern of expression of microfibrillar proteins and elastic fibers between ACL and PT. Elastin was increased in ACL, but fibrillin 1 was increased in PT, whereas vitronectin levels were similar between tissues, suggesting that the composition, structure or regulation of these fibers is different between ACL and PT. Tenascin-C and Tenascin-X are mechanosensitive elastic proteins [72]–[75], and their elevation in ACL compared to PT may reflect differential responsiveness to mechanical loading.

### 4.6 ECM-regulatory proteins

We identified several ECM regulatory proteins not previously associated with tendon or ligament. Histidine-rich glycoprotein and alpha-1-antitrypsin were enriched in ACL compared to PT. Histidine-rich glycoprotein potentiates the action of heparanase by binding to heparan sulfate [76], and the serine protease inhibitor alpha-1-antitrypsin inactivates elastase, binds members of the ADAMTS family and is a substrate for MMP9 [77]–[79]. Alpha-1-microglobulin/bikunin precursor (AMBP) was highly enriched in PT compared to ACL, and tissue levels were similar to or greater than those of albumin. After normalization to albumin, levels of AMBP in females were greater than those in males. AMBP undergoes post-translational modification to alpha-1-microglobulin [80], [81], which can protect collagen fibrils from oxidative damage and up-regulate collagen and elastin genes [80], suggesting either a role for AMBP in protection of PT from injury or of accumulated AMBP in PT over a life-time.

### 4.7 Comparison between Males and Females

Alcohol dehydrogenase 1B was the protein most enriched in females compared to males. Alcohol dehydrogenase 1 isoforms metabolize a range of several biological substrates, including ethanol to acetaldehyde [82], and reduce the aldehyde products of lipid peroxidation [83], thus potentially reducing harmful abnormal accumulation of advanced glycation end products (AGEs), formed from the reaction of lipid-derived aldehyde products with cysteine or lysine residues or proteins [82]. Accumulation of AGEs in tendons increases their stiffness [84], [85], thus enrichment of alcohol dehydrogenase 1B in females could result in differential AGEs accumulation and mechanical properties between male and female.

Complement component 9 was also highly enriched in female ACL compared to male ACL and in ACL compared to PT, and complement C3 was enriched in ACL after normalization to albumin. COMP, PRELP, CHAD, and biglycan were enriched in ACL compared to PT and are known to regulate complement [64], [86], but the interaction between these structural components and complement in ACL remains to be determined.

In previous studies, pooled male and female ACL from a wide range of ages identified expression of TMP-1,-2, -3, and -4, along with expression of MMP -1,-3,-7,-9,-11,-14,-17 and -18 [87]. Previously, greater levels of MMP-3 mRNA expression were identified in female ACL compared to male ACL, and protein levels correlated tightly with mRNA levels [88]. We additionally identified MMP-10 in ACL, and found enrichment of MMP3 in female PT compared to male. TSP1 was enriched in female PT compared to male, and TSP4 was enriched in ACL compared to PT. TSP1 is expressed in tendon [12], and is critical for maintaining TGFβ1 in a high level of activity [89], but this may be detrimental to tenocyte survival after injury [90]. TSP4 expression occurs in both tendon and ligament [12], and is structurally similar to COMP [91], which was also enriched in ACL compared to PT in the current study. Both COMP and TSP4 share a common binding site on the collagen fibril [91] and COMP binding is exercise-responsive, whereas TSP4 immunoreactivity is not [91]. Thus, enrichment of both TSP4 and COMP in ACL may represent a regulatory mechanism for response to exercise.

Myocilin was highly enriched in male compared to female ACL. Its exact function is unknown [92], but it is a member of the olfactomedin domain-containing proteins that may modulate Wnt signaling and regulate the actin cytoskeleton, and it interacts with members of the syntrophins and fibronectin [93]–[95]. In the musculoskeletal system, myocilin regulates muscle hypertrophy and atrophy pathways [93], and is expressed in the cytoplasm of cells in the annulus of the intervertebral disc, where a role for response to dynamic mechanical loading or to TGF-β signaling has been suggested [96]. Therefore, increased myocilin expression in male ACL may suggest differential ability of the male ACL to respond to changes in mechanical loading or growth factor signaling compared to female ACL. Albumin was enriched in males compared to females and this finding is consistent with higher serum albumin in men than in women, although this effect is lost post-menopause [97], [98].

### 4.8 Proteins Related to Tendon or Ligament Disease

Several proteins or pathways implicated in tendinopathy demonstrated differential regulation between PT and ACL and between male and female. Annexin A2 and A2/S100A10 is upregulated in hypoxia [39], and was increased in PT. In ACL, the anti-apoptotic proteins clusterin and annexin A5 were enriched, both of which have previously been identified in tendinopathy [41]. After correction to albumin, clusterinwas enriched in female ACL compared to male. Complement component 9 is a critical part of the terminal membrane attack complex and was enriched in ACL [43]. Periostin is upregulated in tendinopathy [40], and in this study was found to be enriched in ACL. Together, these data suggest that key proteins associated with tendinopathy are differentially enriched between normal ACL and PT.

### 4.9 Blood, Serum and Amyloid Proteins

Several ‘plasma’ or ‘blood’ proteins differentially enriched between ACL and PT were also annotated to ‘ECM-regulators’ by the matrisomal project, and have already been discussed above. Transforming growth factor-beta-induced protein ig-h3 (BGH3) was enriched in female ACL at similar levels to albumin. BGH3 is produced by activated macrophages to stimulate ECM repair mechanisms including collagen accumulation [42]. Together these data suggest enhanced responsiveness of female ACL to remodeling, or accumulation from degenerative processes.

### 4.10 Discussion of Materials and Methods

Tendon and ligament are fibrous tissues that are difficult to disrupt for further digestion or analysis by manipulation of fresh tissue. The method described here of lyophilizing and pulverizing using a freezer mill to prepare samples for proteomic analysis was chosen to maximize the soluble protein yield and minimize the potential for chemical modification of proteins through changes to post-translational modifications, denaturation or proteolysis during processing [99]. Use of devices to pulverize tendon at extremely low temperatures to extract mRNA has been previously reported [100]. Others have used pulverized cartilage samples for proteomic analysis, and in a comparison of manual homogenization and automated deep frozen homogenization and subsequent proteomic analysis of a variety of samples, automated deep frozen homogenization improved protein extraction efficiency, detection, reproducibility of sample preparation, and disruption of membrane-bound intracellular compartments to release soluble proteins [99], [101]. In this study, we elected to perform simple tryptic digestion after pulverization in order to minimize the possibility of peptide modification or disruption caused by other extraction processes and this method was justified by virtually 100% solubilization observed after overnight tryptic digestion.

For several proteins, we observed significant differences by ANOVA and ‘large’ effect sizes estimated by Cohen's d after evaluation of fold-change data (**Tables 1** **and** **2**), but after comparison of quantitative data by Student's T test and Cohen's d (**Table S5** and **Figures 3** **,** **4** **,** **5**), differences were found to be non-significant. There are several possible reasons for this discrepancy: 1) The discrepancy may result from the fact that the fold-change data is calculated using all peptides, but estimation of protein abundance (quantification) was performed based on average intensity of only the two or three ‘best flier’ peptides for each protein compared to the surrogate standard at known concentration (ADH1_YEAST). Collagen peptides are often highly modified, and the ‘best flier’ quantitation approach may result in underestimation of protein content if a peptide which can be modified is chosen for quantification. 2) The different observation between measurement approaches may suggest limited biological powering, despite power analysis performed during experimental design and that the findings of differences for these proteins are interesting but preliminary and not conclusive.

It is important to emphasize that for the current study, we performed the analyses with an emphasis on minimizing type I error and incorrectly rejecting the null hypotheses, given that the results of this study are likely to result in further investigations into the mechanism of differential ACL injury risk between male and female. Nonetheless, these findings highlight proteins that had an interesting fold-change and biological plausibility as “candidate markers”, which will be evaluated in future studies using targeted proteomic methods, including LC/MS/MS using Multiple Reaction Monitoring, which should be considered the ‘gold-standard’ for validating protein expression changes [102].

### 4.11 Study Limitations

One limitation of this study with respect to potential clinical relevance was the age of the donors relative to the age of the typical ACL reconstruction patient. Other studies have identified changes in the composition and structure of the ACL with age [10], but others have found no correlation with age in normal PT [103]. Nonetheless, these data suggest that there are key compositional differences between ACL and PT, and provide new insight as to pathways which may be involved in ACL injury, and potentially in the differential injury response of female ACL. Future work to improve understanding of these differences and to understand the role of these injury pathways is required, particularly in ACL and PT harvested from donors at different ages, and from those at various stages following ACL injury or repair. The methods presented here allow detailed quantitative measurements of the proteome of highly collagenous tissues and thus are potentially applicable to other connective tissues.

MS/MS identifications have been made available as scaffold files, which can be downloaded at the following link: https://discovery.genome.duke.edu/express/resources/2365/Little_MSMS_Supplement.7z.

## Supporting Information

Figure S1 **Total peptide count by 2DLC fraction for all samples and total ion count by 2DLC fraction per sample for anterior cruciate ligament (ACL) and patellar tendon (PT).**
(TIF)

Table S1 **Peptide-level quantitative data for each anterior cruciate ligament (ACL) sample, along with identification scores for each search engine, peptide mass, charge, and retention time.**
(XLSX)

Table S2 **Peptide-level quantitative data for each patellar tendon (PT) sample, along with identification scores for each search engine, peptide mass, charge, and retention time.**
(XLSX)

Table S3 **Summed intensities of all peptides in anterior cruciate ligament (ACL) for each protein by subject and differential abundance.**
(XLSX)

Table S4 **Summed intensities of all peptides in patellar tendon (PT) for each protein by subject and differential abundance.**
(XLSX)

Table S5 **Quantitative values for each protein, in both anterior cruciate ligament (ACL) and patellar tendon (PT) for each sample.** For proteins which were present in both ACL and PT at quantifiable levels, the fold-changes between both PT and ACL and male and female within these tissues and the statistical confidence of these changes are also reported.
(XLSX)
