# Supplementary figures and images for: Proteomic Differences between Male and Female Anterior Cruciate Ligament and Patellar Tendon
Source: PLoS One. 2014 May 12;9(5):e96526. doi: 10.1371/journal.pone.0096526 (PMC4018326; doi:10.1371/journal.pone.0096526)

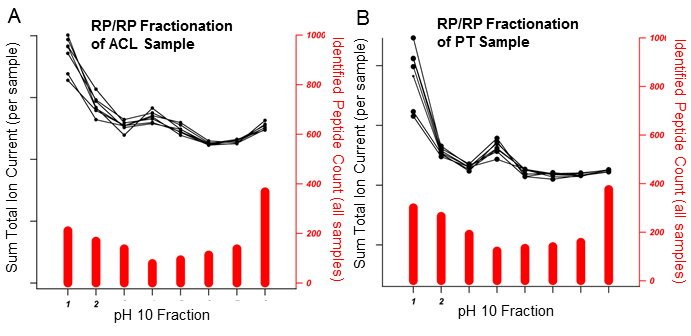

Supplement: Figure S1 — Total peptide count by 2DLC fraction for all samples and total ion count by 2DLC fraction per sample for anterior cruciate ligament (ACL) and patellar tendon (PT). (TIF) [file pone.0096526.s001.tif]
